# *Salmonella* Strains Isolated from Galápagos Iguanas Show Spatial Structuring of Serovar and Genomic Diversity

**DOI:** 10.1371/journal.pone.0037302

**Published:** 2012-05-16

**Authors:** Emily W. Lankau, Lenin Cruz Bedon, Roderick I. Mackie

**Affiliations:** 1 Department of Animal Sciences and College of Veterinary Medicine, University of Illinois at Urbana-Champaign, Urbana, Illinois, United States of America; 2 Armador del Pirata, Puerto Ayora, Isla Santa Cruz, Galápagos Islands, Ecuador; 3 Department of Animal Sciences and Institute for Genomic Biology, University of Illinois at Urbana-Champaign, Urbana, Illinois, United States of America; Indian Institute of Science, India

## Abstract

It is thought that dispersal limitation primarily structures host-associated bacterial populations because host distributions inherently limit transmission opportunities. However, enteric bacteria may disperse great distances during food-borne outbreaks. It is unclear if such rapid long-distance dispersal events happen regularly in natural systems or if these events represent an anthropogenic exception. We characterized *Salmonella enterica* isolates from the feces of free-living Galápagos land and marine iguanas from five sites on four islands using serotyping and genomic fingerprinting. Each site hosted unique and nearly exclusive serovar assemblages. Genomic fingerprint analysis offered a more complex model of *S. enterica* biogeography, with evidence of both unique strain pools and of spatial population structuring along a geographic gradient. These findings suggest that even relatively generalist enteric bacteria may be strongly dispersal limited in a natural system with strong barriers, such as oceanic divides. Yet, these differing results seen on two typing methods also suggests that genomic variation is less dispersal limited, allowing for different ecological processes to shape biogeographical patterns of the core and flexible portions of this bacterial species' genome.

## Introduction

Recent bacterial biogeography studies demonstrate that both niche selection and dispersal limitation influence bacterial distribution patterns [1]–[5]. Dispersal limitation can result in a genetic distance-decay pattern (or isolation-by-distance relationship). Distance-decay is a negative correlation between geographic distance and genetic similarity that presumably results from higher gene flow between populations that are closer together spatially due to more frequent dispersal events (i.e. the converse of gene flow is isolation-by-distance) [2], [5]–[9].

Host-associated bacterial populations could be strongly dispersal limited if host distributions constrain microbial migration potential [3], [10]–[13]. It is not clear if this restriction might possibly relax when host-associated microbes can survive in non-host environments or can associate with multiple host species. For example, gastrointestinal bacteria responsible for food-borne outbreaks can persist for days to months in a variety of environments, include sea water [14]–[20], resulting in wide and rapid dispersal by agricultural distribution systems [21]–[24]. A single strain may quickly travel long distances and thus, would not necessarily be expected to demonstrate clear genetic-by-distance relationships. However, it is unclear how common rapid long-distance dispersal events are in natural host-bacterial systems, or if such events are an artifact of agricultural production systems.

*Salmonella enterica* is a Gram negative proteobacterium that primarily resides in animal gastrointestinal tracts, but can also survive in the environment, food, and water [25]. While *S. enterica* is generally pathogenic to warm-blooded animals, it is rarely reported to cause illness in reptiles [26]–[27]. *Salmonella enterica* ecology in reptile populations is not entirely understood. Field studies suggest that *S. enterica* diversity in reptile populations is dependent on local exposures, with geographically proximate heterospecific populations sharing *S. enterica* strains [28]–[29]. In contrast, in warm-blooded species, *S. enterica* is primarily associated with food-borne outbreaks that may affect large geographic areas [e.g. 22]–[23], demonstrating a potential for rapid, long-distance dispersal.

Island systems have long contributed to understanding ecological and evolutionary effects of geographic isolation [30]–[36]. The Galápagos Islands, approximately 1,000 km off the west coast of Ecuador, have famously contributed to such studies. These islands are host to two ecologically distinct types of large herbivorous lizard, the land and marine iguanas (*Conolophus* species and *Amblyrhynchus cristatus*, respectively). Populations of both genera are present throughout the island chain, with overlapping distributions at some locations [37]. Genetic studies show historical patterns of sequential colonization as the island chain developed, with little or no evidence of significant contemporary gene flow among established populations [38]–[39]. Galápagos iguanas commonly carry *S. enterica* in their digestive tracts and land and marine iguana populations from the same location share site-specific bacterial strains [29]. These findings suggest that this system is a suitable model for exploring the effect of strong host isolation on bacterial population structure in a natural system.

In this study, we ask the question: How does geographic isolation shape *S. enterica* strain diversity and population similarity across host populations? We applied phenotypic strain typing (i.e. serotyping) and genomic fingerprinting to fecal-derived *S. enterica* isolates from Galápagos land and marine iguanas (*Conolophus* species and *Amblyrhynchus cristatus*, respectively) from five sampling sites on four islands. We hypothesized that if geographic isolation is a barrier to enteric bacterial transmission, then each island should be host to a unique strain pool. However, if this barrier is not absolute, then we would expect that bacterial dispersal would be more likely between proximate sites (i.e. distance-decay).

## Methods

### Study design and sampling sites

**Figure 1:**
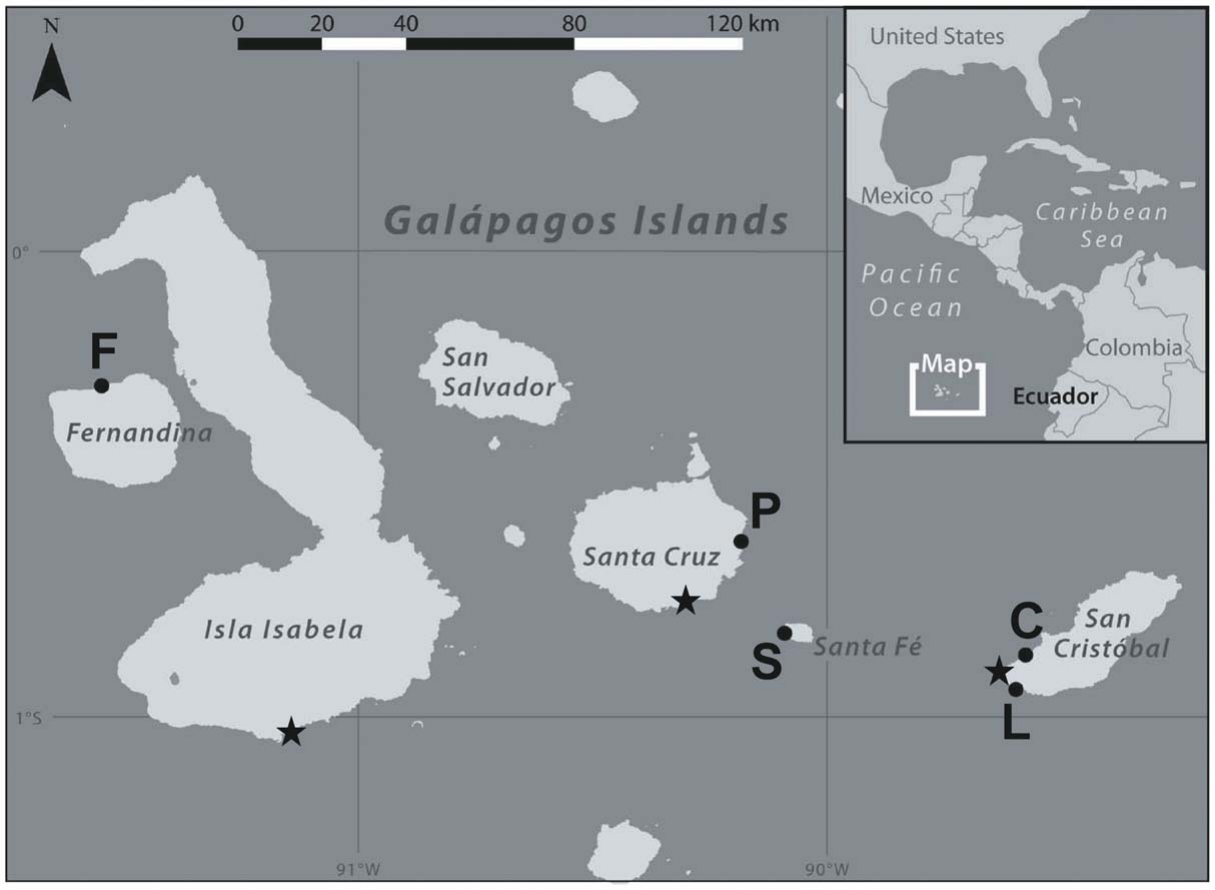
Sampling locations where fecal specimens were collected. Samples were collected from marine iguanas (*Amblyrhynchus cristatus*) on Isla San Cristóbal sites Punta Carola, La Lobería, Isla Santa Fe, Isla Plaza Sur and Isla Fernandina and from two species of land iguanas on three islands (*Conolophus pallidus* on Isla Santa Fe and *Conolophus subcristatus* on Isla Plaza Sur and Isla Fernandina). Black stars indicate the three major port towns of the Galápagos Islands, Puerto Baquerizo Moreno on Isla San Cristóbal, Puerta Ayora on Isla Santa Cruz, and Puerto Villamil on Isla Isabela.

Iguana fecal samples were collected at five sites on four islands of the Galápagos chain (Figure 1, Table 1). Specimens were collected from the ground after deposition using a sterile wooden applicator. We selected fecal samples from different areas of each site and collected samples over a short period of time (24–48 hours) to avoid repeated sampling of the same individual. Fecal specimens were obtained from both iguana types on Isla Plaza Sur (N_land_ = 11, N_marine_ = 12), Isla Fernandina (N_land_ = 6, N_marine_ = 12), and Isla Santa Fe (N_land_ = 12, N_marine_ = 2) and from marine iguanas on Isla San Cristobal (Loberia N = 12, Punta Carola N = 12).

**Table 1:** Sampling site characteristics and *Salmonella enterica* prevalence in Galápagos iguana populations.

Site (Abbr.*)	Island (A km^2^)**	Iguana Species	N	% Pos. (N Pos)	N Serovar	N_serovar_/N_pos_
Punta Carola (C)	San Cristóbal (558)	Marine	12	75.0% (9)	5	0.56
La Lobería (L)	San Cristóbal (558)	Marine	12	75.0% (9)	5	0.56
(whole island) (P)	Plaza Sur (0.13)	All samples	23	65.3% (15)	7	0.47
		Marine	12	75.0% (9)		
		Land∧	11	54.5% (6)		
El Miedo (S)	Santa Fe (24)	All samples	14	42.9% (6)	4	0.67
		Marine	2	50.0% (1)		
		Land$	12	45.5% (5)		
Cape Douglas (F)	Fernandina (642)	All samples	18	44.4% (8)	7	0.88
		Marine	12	50.0% (6)		
		Land∧	6	33.3% (2)		

* Abbr. = Abbreviation; % Pos. = % of samples positive for *S. enterica*; Npos = Number of samples positive for *S. enterica*; N serovar = Number of unique serovars identified in each site; Nservar/Npos = serovar richness for each site adjusted for the number of positive samples, in units of number of serovars/positive sample.

** Land area estimates from [37];

∧ *Conolophus pallidus*;

$ *Conolophus subcristatus*.

Samples were placed in sterile plastic tubes, were stored at 4°C during travel and were transported to Urbana, Illinois for storage at −20°C. Sample collection was performed under an approved animal use protocol (University of Illinois protocol # 90410). Samples were collected with permits from the Galapagos National Park (PNG Autorization de Proyecto PC-21-06 Ext 01-09) and were exported under CITES permit (007-09/PNG).

### Isolation and identification of *S. enterica*

*Salmonella enterica* was isolated from frozen fecal samples using previously published protocols [29], [40]. Briefly, 0.5 g of feces was pre-enriched in buffered peptone water (BPW) at 37°C for 24 hours followed by enrichment in a 1∶9 ml dilution of turbid BPW:Rappaport-Vassiliadis broth (RVB) at 37°C for another 24 hours. Turbid RVB was then streak plated on selective-differential media (Xylose lysine deoxycholate). Plates demonstrating no *Salmonella*-characteristic growth at 24 hours were re-incubated for an additional 24 hours before being considered negative. These samples were retested once from the original fecal samples before being considered negative for *S. enterica*.

Presumptive *Salmonella* isolates were then tested on lysine iron agar and triple sugar agar to confirm membership in the genus. Up to three confirmed *Salmonella* isolates were frozen at −70°C in 40% glycerol for further analysis.

### Serotyping and genomic fingerprinting

Traditional serotyping and genomic fingerprinting have been previously used to describe *S. enterica* strain patterns in this system [29]. Serotyping is a phenotypic bacterial typing approach which identifies O (outer membrane) and H (flagellar) antigenic variation among strains [41]. Observed concordance between sequence-based genotyping and serotype identity suggests that serotyping is a strong phenotypic marker for genetic strain relationships [29], [42]–[43]. One isolate per sample was submitted for serotyping at the National Veterinary Service Laboratory, Ames, IA, USA.

While serotyping is often sufficient for drawing epidemiological links, higher resolution typing of *S. enterica* strains is often desirable to better understand outbreak dynamics. Genotyping techniques which target genomic-level variation have proven most successful at differentiating very closely related strains of *S. enterica* [44]–[46]. Repetitive extrapalendromic PCR (Rep-PCR) produces genomic fingerprints through amplification of intervening sequences between repetitive elements of the bacterial genome, resulting in numerous bands of varied size that can be separated by agarose gel electrophoresis. Rep-PCR has been shown to be both highly repeatable and highly reliable for differentiation of *Salmonella* isolates [46].

Up to three isolates per sample were analyzed by Rep-PCR. DNA was extracted from colonies grown on tryptic soy agar using a modification of the manufacturer's protocol for Instagene chelex resin (Biorad, Hercules, CA, USA). Two to four large, well-isolated colonies were placed in 100 µl of 6% chelex 100 resin and lysed at 95°C for 10 minutes. Lysates were centrifuged for 5 minutes at 14000 g and 80 µl of supernatant were transferred to a new storage tube. Rep-PCR was performed as in previously described protocols using primers ERIC1R (5′-ATG TAA GCT CCT GGG GAT TCA-3′) and ERIC2 (5′-AAG TAA GTG ACT GGG GTG AGC G-3′), targeting enterobacterial repetitive intergenic consensus (ERIC) repetitive motifs [47]–[48]. PCR was performed on a TGradient thermocycler (Biometra, Germany) with an initial denaturation at 95°C for 2 minutes, followed by 30 cycles each of 94°C for 3 seconds, 92°C for 30 seconds, 50°C for 1 minute and 65°C for 8 minutes, and a final extension at 65°C for 8 minutes. Polymerase chain reaction amplification mixtures (25 µl) included 1.75 U of Takara Taq polymerase (Takara Bio Inc., Japan), 1× Takara PCR Buffer with 2.5 mM (final concentration) of MgCl_2_, 2.0 mM Takara dNTP mixture (0.5 mM each), 1 µM each of the forward and reverse primers, and approximately 50 ng of template. PCR products were separated on a 2% Agarose gel in 0.75% TAE (Tris-Acetate EDTA) run at 80 V for 12 hours. Three lanes of a 1 kb plus DNA ladder (Novartis, Carlsbad, CA) were included to allow for standardization of molecular weight assignments to DNA fragments, with one lane at each end of the gel and one located in the approximate middle to allow for correction of irregular gel runs. Gels were stained with ethidium bromide and digitally photographed using an AlphaImager ™ 2200 (Alpha Innotec Co., San Leandro, CA, USA).

### Statistical analysis

Rep-PCR band assignment and sizing from gel images was done using BioNumerics version 4.0 (Applied Maths, Belgium). Band assignments were exported as a binomial presence-absence matrix for statistical analysis. To control for non-independence (i.e. pseudoreplication) resulting from inclusion of multiple isolates per individual iguana fecal sample, fingerprint profiles of isolates from the same individual iguana were nested by averaging presence/absence within an individual host for analysis (see online supplemental information for [29]).

To evaluate explanatory variable effects on bacterial population structure (sampling site, host genus, and geographical coordinates of site), we used the adonis function in the vegan package in R [49] to perform a series of permutation (non-parametric) multivariate analysis of variance tests on genomic fingerprint patterns. These models are similar to more traditional multivariate analysis of variance (MANOVA), but they partition the sums of squares of distance matrices among treatments and have relaxed assumptions relative to traditional MANOVA. For categorical variables, a significant p-value indicates that isolates within a group are more similar to others within the group, relative to isolates from other groups. For a continuous variable, such as geographic coordinates, a significant p-value indicates that more proximate isolates are more similar (i.e. is analogous on a multivariate level to distance-decay). Significance in the permutation tests was determined by comparing the observed effects against 5000 random permutations of the data for each model run independently. Interaction terms were not included as land iguanas were only present for sampling at 3/5 sites.

Geographical location was included in subsequent permutation MANOVA models as the latitude and longitude coordinates of each site or as a rotated axis derived from the principal component scores of the sites' latitudes and longitudes. This ordination produced two orthogonal axes oriented along a southeastern to northwestern axis, PC1, and along a northeastern to southwestern axis, PC2. Many plant and animal species in the Galápagos demonstrate a southeast-to-northwest genetic diversity gradient, with the most divergent and isolated populations located on the older islands in the eastern and central islands and younger, more genetically recent populations in the northwestern sites (i.e. the “progression hypothesis”) [38]–[39], [50]. Thus, this principal components rotation of the primary latitudinal and longitudinal axes has the potential to more fully capture the primary geographical pattern in this system by capturing the majority of the variation present in the geographical axis into a single variable (PC1).

We then performed multivariate ordinations on the genomic fingerprint patterns to visualize the significant effects detected by permutation MANOVA, using both an unconstrained and a constrained correspondence analysis (CA and CCA, respectively, both performed with the cca function in the vegan package) [49]. Correspondence analysis is a statistical ordination technique that summarizes multivariate data, such as banding patterns, into two-dimensional space for visualization of patterns in the data. When unconstrained, CA can provide a visual demonstration of dominant patterns in a dataset, independent of other variables of interest. When constrained by a variable of interest, CCA can provide a visual summary of patterns in the data that specifically relate to that variable. CCA was performed using sampling site as the constraining variable, as this factor explained the largest amount of variation (i.e. had the highest R^2^) in the various permutation MANOVA analyses. The CCA isolates those aspects of the genomic information most strongly contributing to geographic structuring. We then performed an environmental fitting of the previously described PC1 and PC2 location axes on the CA and CCA patterns using the envfit function in the vegan package of R [49]. A significant p-value on environmental fitting indicates that there is a statistically significant association between the variable of interest and the summary of the data provided by the correspondence analysis ordination; the environmental fit can be plotted on the ordination graph to demonstrate the nature of this relationship visually.

Basic statistical and graphing procedures were performed in either JMP 8 (SAS Institute, Cary, NC) or R statistical language [51].

## Results

### Prevalence and serovar patterns

Prevalence of *S. enterica* in Galápagos iguana populations ranged from 33% to 75%. Marine iguanas tended to have higher prevalence (68%, n = 50) than land iguanas (45%, n = 29) but this difference was only marginally statistically significant (Fisher exact test p = 0.058, Table 1). *Salmonella enterica* prevalence was not significantly different among sites within a host type (Fisher exact tests: marine iguana among sites p = 0.544, land iguana among sites p = 0.702).

We isolated 23 unique serovars from 47 iguanas distributed among the five sampling sites (Table 2, serogroups as in [52]). A number of serovars detected in this study were also noted in a previous sampling of a subset of these sites in 2005 (Table 2). Serovar richness controlled for the number of positive samples varied among sites, ranging from 0.47–0.88 serovars/positive sample (Table 1).

**Table 2:** Sampling site distributions of *Salmonella enterica* serovars isolated from Galápagos land and marine iguanas in 2005 and 2009.

				Sites** 2009						Sites 2005&		
	Sub-species	Serotype	Serogroup*	Total	C	L	P	S	F	Total	P	S
**2009 & 2005**	I	Muenchen	O:8 (C2–C3)	2	1		1					
	I	Manhattan	O:8 (C2–C3)	2			2			2	2	
	I	Sandiego	O:4 (B)	6			6			10	7	3
	I	Poona	O:13 (G)	3			3			2	2	
	IV	53:z4,z23-	O:53	1			1					
	IV	44:z36-	O:44	1			1					
	I	Pomona	O:28 (M)	4			1	3		3	2	1
	I	Berta	O:9 (D1)	4		3		1				
	I	Treforest	O:51	1				1				
	I	Rough O:L,V:1,7	R	2				1	1	3		3
	I	Montevideo	O:7 (C1)	2	2					1		1
	I	Bredeney	O:4 (B)	1	1							
	I	Newport	O:8 (C2–C3)	2	2							
	II	II 47:b:1,5	O:47 (X)	3	3							
	I	Mjordan	O:30 (N)	2		2						
	I	Saintpaul	O:4 (B)	1		1						
	I	Reading	O:4 (B)	2		2						
	I	Rubislaw	O:11 (F)	1		1						
	I	Rough O:y:1,7	R	1					1			
	I	57:b:-	O:57	2					2			
	I	Rough O:c:enz15	R	1					1			
	I	Wedding	O:28 (M)	1					1			
	IV	Rough O:g,z,51:-	R	1					1			
	Untyped$	Untyped	Untyped	1					1			
**2005 only**	I	Panama	O:9 (D1)							6	6	
	I	Oranienburg	O:7 (C1)							2	2	
	I	Rough O:z10:enx	R							1		1
	I	Rough O:z10:enz15	R							3		3
	I	SSI 28:v:-	O:28 (M)							1		1
Total N				47	9	9	15	6	8	34	21	13

* Serogroups as in [52].

** C = Punta Carola, Isla San Cristóbal, L = La Loberia, Isla San Cristóbal, P = Isla Plaza Sur, S = Isla Santa Fe, F = Isla Fernandina.

$ untyped isolate established as presumptive *Salmonella* spp. by metabolic assay but submission to the National Veterinary Services Laboratory was found to be contaminated and was not typed further.

& *S. enterica* isolates from 2005 were part of a previous study and are described further in [29].

### Genomic fingerprint patterns

**Figure 2:**
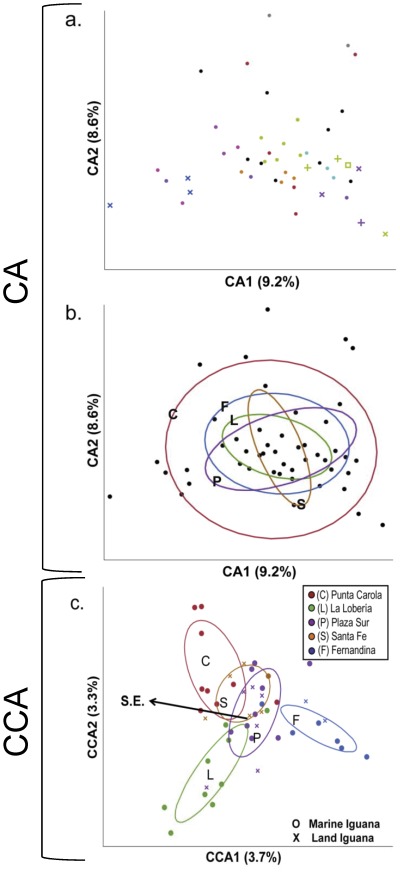
Unconstrained and constrained correspondence analysis of *Salmonella enterica* genomic fingerprints. Panel a) presents the unconstrained correpondance analysis (CA) with O antigen serogroups represented by color and serovars within these groups with differing symbols. Panel b) presents the same ordination (CA), but with 50% contour ellipses for sites. Environmental fitting of PC1 and PC2 geographic location axes to this unconstrained ordination were not significant (PC1 environmental fit R^2^ = 0.004, p = 0.92; PC2 environmental fit R^2^ = 0.054, p<0.282). Panel c) presents the same data analyzed by correspondence analysis constrained by sampling site (CCA, including 50% contour ellipses for sites). Environmental fitting of PC1 and PC2 geographic location axes explains a significant portion of the variation in this ordination (PC1 environmental fit R^2^ = 0.678, p<0.001; PC2 environmental fit R^2^ = 0.027, p<0.538; S.E. indicates the cardinal direction southeast).

Genomic fingerprint patterns on unconstrained ordination reflected serogroup level differences similar to those seen on serotyping (Figure 2a, CA). Grouping of isolates by sampling site is not notable in this unconstrained analysis (Figure 2b, CA). However, sampling site was a significant factor on permutation MANOVA analysis of genomic fingerprints (Table 3, site R^2^ = 0.137, p = 0.004). In models including sampling site, geographic factors latitude, longitude and PC2 were not significant. However, PC1 was a significant factor explaining genomic similarity among isolates (Table 3). In a model including site, PC1, and PC2, both site and PC1 significantly explained aspects of genomic variation. This suggests that while sites differed uniquely from each other (as seen also in the serotyping results in Table 2), there was also a portion of genomic variation structured along this geographical axis. PC1 was also a significant environmental fit to the sampling site-constrained canonical correspondence analysis, aligning directionally in a southeast to northwest direction across sampling sites (Figure 2c; PC1 environmental fitting R^2^ = 0.678, p<0.001).

**Table 3:** Permutation MANOVA analysis of rep-PCR fingerprint patterns, evaluating the influence of site, and geographic location on *Salmonella* similarities.

Source	F	R^2^	p
Site	1.687	0.137	0.004**
Host	1.163	0.024	0.306
Site	1.666	0.137	0.004**
Latitude	1.181	0.024	0.323
Longitude	1.053	0.022	0.371
Site	1.698	0.070	0.018**
PC1 (S.E.-N.W.)	2.176	0.045	0.010**
PC2 (S.W.-N.E.)	1.072	0.022	0.381

* Significant at α = 0.10;

** Significant at α = 0.05.

## Discussion

Wild reptile populations commonly harbor diverse *S. enterica* assemblages. In this study, adjusted serovar richness (i.e. N serovars/positive sample) was comparable to that seen in previous studies (the average for 8 published studies = 0.44 serovars/positive sample with a range of 0.19–0.75 serovars/positive sample) [28]–[29], [40], [53]–[57]. *Salmonella enterica* is a common and diverse member of the enteric biota of Galápagos land and marine iguanas on multiple islands [29], [57]–[59]. This diversity presented a unique opportunity to more fully explore the effects of host population isolation on strain and genetic diversity patterns of this host-associated enteric bacterium.

The observed pattern of distinct serovar assemblages at each site might suggest that rare and stochastic strain dispersal among sites is a driving force for determining *S. enterica* population structure. When dispersal limitation is a primary driver of population or community composition, considerable site-to-site variation occurs as species (or strains) arrive and establish by chance at each site independently [60]–[61].

In contrast, when assembly of diversity is driven by niche selection, similar communities should be found in similar selective environments [62]–[64]. While land and marine iguanas represent quite dissimilar selective environments for enteric microbial communities due to host dietary differences [65], *S. enterica* populations of sympatric land and marine environments have been shown to overlap considerably [29]. This suggests that despite host-level differences in enteric environments, local exposures are key for serovar diversity and supports the contention that isolation rather than selection primarily determines the serovar distributions seen in this study.

Also notable is the degree of overlap in serovars detected in 2005 and 2009 iguana fecal samples collected at Isla Plaza Sur and Isla Santa Fe, including Manhattan, Panama, Poona and Sandiego for Isla Plaza Sur and Pomona and Sandiego for Isla Santa Fe. In addition, a local *S. enterica* prevalence study of land iguanas from cloacal swabs collected in 2003 from Isla Santa Cruz, quite near Isla Plaza Sur, also detected serovars Manhattan, Pomona and Poona [57]. This strain stability within geographic locations over a relatively long time period (for ecological processes to influence microbial strain turnover, that is) might suggest that within-site strain selection may also play an important role in maintaining these unique strain pools within local reptile host communities or that immigration of new serovars is rare, resulting in slow serovar turn-over.

While the presence of unique serovar assemblages at each site suggests dispersal is quite rare among sites, genomic patterns were more complicated. On unconstrained ordination (correspondence analysis) of genomic fingerprint patterns, isolates from the same serovar or O-antigen serogroup are generally located in proximate ordination space. A quantitative test of this hypothesis would be difficult with this diverse strain assemblage, but qualitatively this might suggest that genetic relationships among these serovars represent a deeper evolutionary strain history, rather than a more recent ecological distribution of strains among these sites. Sequence-based genotyping methods such as multi-locus sequence typing are generally concordant with phenotypic serotyping but cannot distinguish variation within a serovar [66]–[67]. Thus, the lack of clustering by site in unconstrained analysis is still compatible with conclusions drawn from serovar patterns and is consistent with the significant site effects noted on permutation analysis that were independent of geographical location (i.e. independent of latitude and longitude, or PC1 and PC2 axes).

Yet we also detected a significant southeast to northwest geographic relationship among *S. enterica* populations, even when controlling for these site effects. This suggests that not only are sites significantly unique in strain composition (as detected on both serovar analysis and in the significant site effect on genomic variation), but also that a separate portion of the genomic variation is specifically oriented along this geographical axis. These findings together suggest that while dispersal of specific strains of *S. enterica* among sites is rare, some aspect of *S. enterica* genetic diversity does move among sites and this dispersal is more frequent among proximate sites. Given the considerable horizontal gene transfer often detected in bacterial communities, both generally (as reviewed in [68]–[70]), and within marine iguana intestinal communities [71], it is possible that these directional geographical relationships among *S. enterica* populations are driven by movement of genetic elements among strains. Thus, strains within and between sites may share certain genomic elements, despite not sharing a recent strain history.

This potential for disconnect between evolutionary and ecological history based on different typing methods was hinted at in a previous work in this system [29]. Further, this finding is not inconsistent with current knowledge of *S. enterica* genetic diversity. *Salmonella enterica* shares a large portion of its genome with related bacteria such as *Escherichia coli*, but has in addition the tendency to flexibly gain and lose large, mobile pathogenicity islands [72]. Full genome sequence comparisons of diverse *S. enterica* serovars reveals that genetic differences among strains is primarily based on insertions and deletions of these chromosomal elements, rather than on coding region sequence divergence of the types of genes typically targeted for sequence analysis [73]. Such discordance between genetic markers has also been observed for *Escherichia coli*, where multilocus enzyme electrophoresis of *E. coli* reference strains demonstrated six distinct phylogenetic groups while rep-PCR based analysis grouped these same isolates primarily by species of origin [74].

If serovar (as a proxy for genetic strain history) and genomic fingerprint marker variation operate under different ecological and evolutionary pressures, then this would explain the observed disjunction in biogeographical patterns seen in this study. Given this tendency to flexibly acquire genomic elements, gains and losses of these elements may contribute to differences in genomic fingerprint patterns on a site-to-site basis. These mobile elements may represent *S. enterica-* or Enterobacteriaceae-specific genetic elements or more broadly disseminated transposable elements (e.g. transposons, viruses etc.) horizontally acquired from a wide diversity of bacterial interactions within the enteric or environmental habitat.

Even with this explanation, what is particularly striking about the detected site differences by genomic fingerprinting is the geographical directionality of the site similarity pattern, which correspond to ecological forces unique to this island chain. The Cromwell and Humboldt currents and associated trade winds sweep across the Galápagos Islands in a primarily east-west or southeast-northwest direction for much of the year, and may potentially carry with them a variety of bacterial elements. Thus, the expectation of simple isolation-by-distance patterns may not be the most appropriate test for dispersal-limitation in other similarly structured systems where dispersal capacity may differ by direction.

Visualizing the ecological complexity of *S. enterica* biogeography in this system was dependent on using more than one measure of diversity. Traditionally, biogeographical studies of plants and animals have relied on such combined approaches to fully explore how migration and selection combine to shape geographic patterns. In particular, it is often important to evaluate both neutral and trait-based markers as their geographic patterns will reflect different driving forces (stochastic versus deterministic) [75]–[79]. Given the growing understanding of how horizontal gene transfer influences bacterial genetics, it is a reasonable parallel to suggest that bacterial biogeography might also benefit from simultaneous measures of core and mobile genetic diversity [80]–[82].

These results also suggest that diversity patterns arising from dispersal limitation may be more subtle than simple isolation-by-distance if dispersal potential is asymmetrical [83]. However, simple isolation-by-distance certainly remains a reasonable initial model for consideration (as reviewed in [84]). Only a few studies to date have begun to incorporate potential asymmetries in dispersal potentials into understanding microbial population or community biogeography [85]–[86].
